# Posterior Endoscopic Cervical Decompression in Metastatic Cervical Spine Tumors: An Alternative to Palliative Surgery

**DOI:** 10.5435/JAAOSGlobal-D-22-00201

**Published:** 2022-11-02

**Authors:** Vit Kotheeranurak, Khanathip Jitpakdee, Yodsawee Pornmeechai, Yutthana Khanasuk, Panyajarn Laohapornsvan, Jin-Sung Kim, Wongthawat Liawrungrueang

**Affiliations:** From the Department of Orthopaedics, Faculty of Medicine, Chulalongkorn University, King Chulalongkorn Memorial Hospital, Bangkok, Thailand (Dr. Kotheeranurak); Center of Excellence in Biomechanics and Innovative Spine Surgery, Chulalongkorn University, Bangkok, Thailand (Dr. Kotheeranurak); the Department of Orthopedics, Queen Savang Vadhana Memorial Hospital, Chonburi, Thailand (Dr. Jitpakdee, Dr. Pornmeechai, and Dr. Khanasuk); Ratchapipat Hospital, Medical Service Department, Bangkok Metropolitan Administration, Bangkok, Thailand (Dr. Laohapornsvan); the Department of Neurosurgery, Seoul St. Mary's Hospital, Spine Center, College of Medicine, The Catholic University of Korea, Seoul, South Korea (Dr. Kim); and the Department of Orthopaedics, School of Medicine, University of Phayao, Phayao, Thailand (Dr. Liawrungrueang).

## Abstract

Metastatic spinal cord compression of the cervical spine is a well-known consequence of cancer that generally manifests as an oncological emergency. This study presents and describes an alternative to the minimally invasive posterior full-endoscopic approach for direct decompression and tumor debulking from the metastasis of hepatocellular carcinoma (HCC) in the cervical spine. A 54-year-old man presented with progressive cervical radiculopathy that had persisted for 3 months. The underlying disease was HCC. Radiographic examination revealed evidence of metastatic spinal cord compression with an epidural mass at the C4-C5 levels, which compressed the C4-C5 spinal cord without bony destruction. The modified Tomita score was 6 to 8 points based on palliative surgery. A posterior full-endoscopic approach to remove the tumor from the metastasis of HCC in the cervical spine was done. A postoperative radiographic study revealed adequate tumor mass resection and spinal decompression. The patient was extremely satisfied with this alternative treatment and achieved complete neurologic recovery at 1 month and no recurrent symptoms at the 6-month follow-up. The technique of posterior full-endoscopic decompression of cervical metastasis causing unilateral radiculopathy, presented in this study, is feasible. This surgical intervention seems to be optional minimally invasive and acts as an alternative to palliative surgery.

The third most prevalent location of malignant neoplasm metastasis in the internal organs is the spine.^1^ The clinical presentation ranges from asymptomatic bony deposition to aggressive spinal cord compression.^2^ Up to 40% of patients with malignant neoplasms acquire spinal metastases during the course of their disease. Approximately 5% to 15% of these individuals suffer from metastatic spinal cord compression.^3,4^ The thoracic and lumbar spine are the most prevalent locations of vertebral metastases when compared with other parts of the vertebral column, and cervical spine metastasis is relatively infrequent, accounting for less than 10% of all spinal metastases.^5^

The primary areas of metastatic deposition are within the bony structure of the spine, with occasional extension into the epidural space, potentially compromising neural structures such as nerve roots or the dural sac. Intradural metastasis is relatively rare, accounting for less than 5% of all spinal metastases.^6^ Nerve compression is a result of physical compression or instability. This results in radiculopathy if the individual nerve roots are compromised. However, in the setting of spinal cord compression, the ensuing myelopathy can be devastating and potentially lethal. In such settings, timely diagnosis and treatment are key prognostic factors.^6,7^

It is difficult to predict the survival of patients with metastatic neoplastic malignancies of the spine. The utility of many grading systems for predicting long-term prognosis was investigated.^8^ The modified Tomita score system predicts patient survival and response to treatment. A modified Tomita score of 6 to 8 points suggests palliative surgery or palliative radiation therapy. The goals of palliative surgical treatment for spinal metastasis are to free the neural structure from ongoing compression by the tumor, stabilize the spinal column in cases of instability, reduce pain, and promote ambulation.^9^ Thus, careful decisions must be made regarding the use of surgical treatment since the open surgical techniques are associated with complication rates of up to 50%.^10^ Therefore, minimally invasive techniques that can adequately decompress the neural structure without affecting spinal stability are beneficial in this group of patients.

We report a case of cervical spine metastasis compressing the spinal nerve roots, causing radiculopathy and neurologic deficit. The patient was treated successfully using posterior endoscopic cervical decompression, a contemporary minimally invasive surgical technique.

## Case Presentation

**Figure 1:**
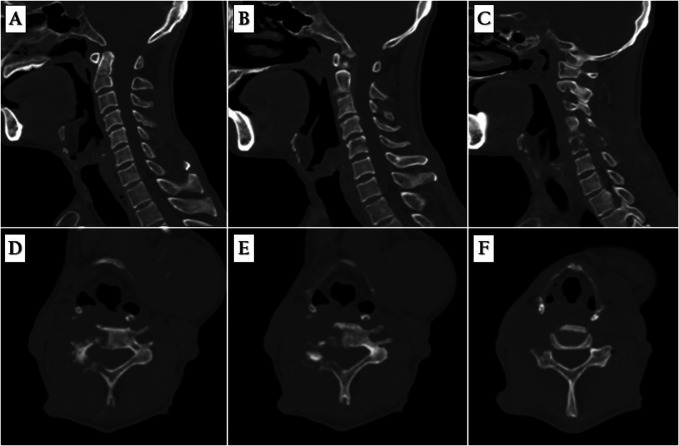
**A**, Mid-sagittal and (**B**) left parasagittal CT scan shows no vertebral destruction. **C**, Right parasagittal CT scan shows osteolytic lesion at C5 vertebral body. **D** and **E**, Axial CT scan shows the osteolytic lesion right transverse process and right pedicel at the C5 level and (**F**) without abnormal lesion at the C6 level.

**Figure 2:**
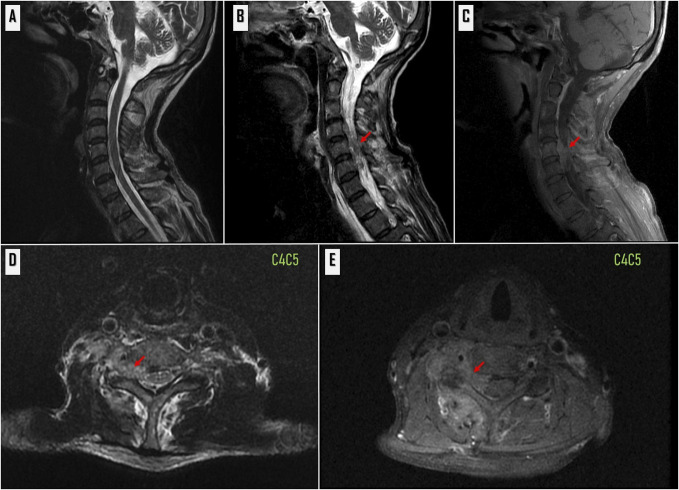
**A**, Mid‐sagittal T2‐weighted MRI shows no vertebral destruction. **B**, Right parasagittal T2‐weighted MRI and (**C**) T1‐weighted MRI withfat saturation and contrast demonstrate the located epidural mass posterior to the spinal cord and compression nerve root at the levelof C4‐C5(Red arrows). **D**, Axial T2‐weighted MRI and (E) T1-weighted with fat saturation and contrast MRI show the lesion located in the right posterior epidural space at the C4-C5 level.

A 54-year-old man with a history of hepatocellular carcinoma (HCC) presented with complaints of progressive severe radicular pain and weakness for 1 month. The patient had undergone chemotherapy for the treatment of HCC. A physical examination revealed a normal mental status and vital signs. The patient had limited neck motion and severe right radicular pain down to the shoulder, with right C5 weakness (motor power grade II). According to clinical measurement scores, the neck disability index was 47, the arm's visual analog scale (VAS) was 7, and the neck's VAS was 3. Special examinations revealed abnormalities in the Spurling test. A rectal examination revealed a normal sphincter tone. Radiographic examination using the CT scan showed the osteolytic lesion right transverse process and right pedicle at the C5 level (Figure 1), and MRI showed an infiltrative enhancing marrow lesion involving the C5 vertebral body, right transverse process, right pedicle, and right lamina without bony destruction. Abnormal soft tissue involving the paravertebral region, right C4/C5 neural foramina with epidural extension along the lateral aspect of the spinal canal at C4-C5 levels, causing compression/involvement of the right C5 root and mild pressure effect on the right-sided spinal cord with a normal spinal cord signal, was noted (Figure 2). MRI results suggested a metastatic epidural mass, as well as a likely diagnosis of a metastatic neoplastic tumor from HCC. His modified Tomita score was between 6 and 8 points, suggesting palliative surgery. C4-C5 was chosen for the full-endoscopic decompression of the cervical metastasis for resection of the epidural mass.

## Surgical Technique

**Figure 3:**
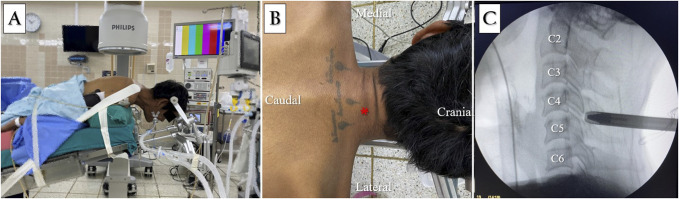
**A**, Photograph showing patient's prone position and stabilization head with the Mayfield holder. **B**, Skin entry point at the lateral to midline 1 cm at the C4-C5 level. **C**, The dilator and working sleeve were placed under fluoroscopic guidance.

**Figure 4:**
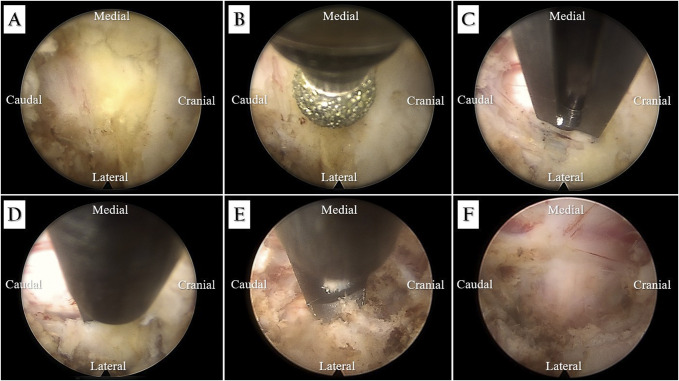
**A**, Diagram showing interlaminar V-point of C4-C5. **B**, A diamond burr and (**C**) Kerrison rongeur were used to perform a laminotomy on the right inferior lamina of C4 and right superior lamina of C5. **D**, Ligamentum flavum resection, (**E**) epidural mass removal, and (**F**) final decompression.

The patient was placed in the prone position on a radiolucent table with a stabilization head for cervical flexion using a Mayfield holder (Figure 3A). The surgical access was found using anatomic landmarks, and the skin incision was marked with the guidance of posteroanterior fluoroscopy. The skin entry point was determined from the lateral to midline, 1 cm at the C4-C5 level, and a stab incision was created (Figure 3B). The dilator was placed under posteroanterior and lateral fluoroscopic guidance (Figure 3C). A dilator was used to advance the working sleeve with a bevelled opening to the C5-C6 level, after which the dilator was removed. The working sleeve was then passed through a cervical endoscope (Richard Wolf GmbH). Between C4 and C5, the interlaminar space (interlaminar V-point) was identified and visible (Figure 4A). An endoscopic diamond burr and a Kerrison rongeur were also used to perform a laminotomy over the right inferior lamina of C4 and the right superior lamina of C5 (Figure 4, B and C). Endoscopic cautery was used to control bleeding after the ligamentum flavum was resected (Figure 4D), and an abnormal epidural tumor was eradicated from the spinal cord and nerve root (Figure 4E). The abnormal epidural mass was removed and sent for pathologic examination. The nerve root tension was measured and evaluated using a nerve hook for adequate spinal decompression (Figure 4F). Active bleeding was monitored and stopped. No drain was placed. The stab incision was closed using absorbable suture 3-0 with interrupted subcutaneous suturing.

## Results

**Figure 5:**
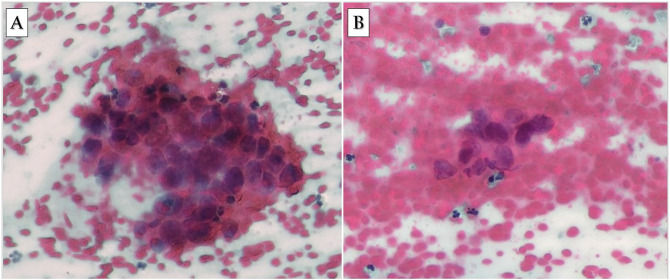
**A**, Photomicrograph (H&E stain ×400) showing the smears show mixed inflammatory cells and predominantly lymphocytes in a bloody background. **B**, A typical cells display enlarged irregular nuclei and hyperchromatin. H&E = hematoxylin-eosin

No intraoperative or postoperative complications were noted. A pathologic study reported mixed inflammatory cells, predominantly lymphocytes, on a bloody background (Figure 5A). Atypical cells displayed enlarged irregular nuclei and hyperchromatin (Figure 5B), which favored metastasis from HCC. The patient received multidisciplinary postoperative care, a rehabilitation program, and proper postoperative concurrent radiation therapy and chemotherapy. Immediate postoperatively, the neck disability index was decreased to 22, arm's and the neck's VAS was 1. The patient's radicular symptoms improved satisfactorily compared with the preoperative symptoms. The patient had near-complete neurologic recovery at 1 month and no recurrent symptoms at the 6-month follow-up, after which he died.

## Discussion

To the best of our knowledge, there have been no previous reports on the detailed surgical technique of the full-endoscopic method to decompress cervical spine metastasis through the posterior approach. A posterior approach was used to decompress the neurologic structures owing to the compressive pathology on the dorsal aspect of the cervical vertebra. Cervical spine metastases with compression of the spinal cord is a serious disorder that can have fatal or life-threatening effects if treatment and diagnosis are neglected. Less than 10% of all spinal metastases involving the cervical spine can cause severe neurologic dysfunction. Therefore, early surgical decompression is typically advised to prevent major consequences.^11^

Surgical intervention and management have considerable morbidity; thus, surgeons frequently have to balance the benefits and risks while treating patients with spinal metastases. Surgical decompression has a risk of complications in individuals with bleeding, prolonged surgery, cervical instability, postoperative wound problem, and other systemic problems.^12^ However, the previous study on minimally invasive spinal decompression procedures has been established in the literature.

Limited open decompressive procedures and direct lateral approaches are generally recognized as minimally invasive techniques for spinal metastases.^13^ When compared with open surgery, minimally invasive surgery is known for having lower risks, blood loss, transfusion rates, and hospital stays, as well as a similar rate of neurologic recovery.^13,14^

In 2001, McLain^15^ reported a case series of thoracic spinal metastases treated with endoscopic decompression. Gao et al^16^ also reported a case of metastasis at the lumbar level that was successfully decompressed with endoscopy by the transforaminal approach. Although percutaneous endoscopy has been used for posterior cervical decompression, we are not aware of any previous reports on the use of this technique for the treatment of cervical metastasis.^15,17,18^

Open laminectomy may cause postoperative instability requiring instrumentation, leading to increased operating time, more extensive muscle dissection, and limited cervical motion.^19^ However, the full-endoscopic decompression technique preserved cervical motion while minimizing blood loss, shortening the surgical operation, and allowed early postoperative ambulation.^20^ The adequacy of decompression is also an important factor. As per the single-level decompression, we were able to visualize the posterior elements clearly with endoscopic instruments, thus leading to successful decompression. Moreover, the continuous fluid irrigation of endoscopic system can help improve the visualization field when compared with limited open decompressive or tubular surgery.

Early recovery is another key advantage of using an endoscope for surgical decompression of cervical metastases. Many patients with cancer are frail and may have a poor prognosis because of the disease process. These patients are unable to tolerate prolonged surgery including corpectomy, open laminectomy, and instrumentation.^21^ As such, minimally invasive endoscopic procedures represent the smallest possible surgical footprint and thus could be a great asset in managing patients with single-level spinal metastasis. However, the possible limitations of this treatment option include availability of the endoscopic system, extensive cervical metastasis involving multiple vertebrae and adequacy of decompression.

This case of metastatic spinal cord compression of the cervical spine is an aggressive neoplastic disease that involves challenging preoperative surgical treatment. In this case, the treatment resulted in a successful outcome. The use of posterior full-endoscopic decompression of cervical metastases in conjunction with tumor debulking has been linked to good results and outcomes.

## Conclusions

The technique of posterior full-endoscopic decompression of cervical metastasis causing unilateral radiculopathy, presented in this study, is feasible. It yields satisfactory results and outcomes and represents the smallest surgical footprint available at present.
